# Assay for dual cargo sorting into endoplasmic reticulum exit sites imaged by 3D Super-resolution Confocal Live Imaging Microscopy (SCLIM)

**DOI:** 10.1371/journal.pone.0258111

**Published:** 2021-10-01

**Authors:** Sofia Rodriguez-Gallardo, Kazuo Kurokawa, Susana Sabido-Bozo, Alejandro Cortes-Gomez, Ana Maria Perez-Linero, Auxiliadora Aguilera-Romero, Sergio Lopez, Miho Waga, Akihiko Nakano, Manuel Muñiz

**Affiliations:** 1 Department of Cell Biology, University of Seville and Instituto de Biomedicina de Sevilla (IBiS), Hospital Universitario Virgen del Rocío/CSIC/Universidad de Sevilla, Seville, Spain; 2 Live Cell Super-Resolution Imaging Research Team, RIKEN Center for Advanced Photonics, Saitama, Japan; Institut Curie, FRANCE

## Abstract

Understanding how in eukaryotic cells thousands of proteins are sorted from each other through the secretory pathway and delivered to their correct destinations is a central issue of cell biology. We have further investigated in yeast how two distinct types of cargo proteins are sorted into different endoplasmic reticulum (ER) exit sites (ERES) for their differential ER export to the Golgi apparatus. We used an optimized protocol that combines a live cell dual-cargo ER export system with a 3D simultaneous multi-color high-resolution live cell microscopy called Super-resolution Confocal Live Imaging Microscopy (SCLIM). Here, we describe this protocol, which is based on the reversible ER retention of two *de novo* co-expressed cargos by blocking COPII function upon incubation of the thermo-sensitive COPII allele *sec31-1* at restrictive temperature (37°C). ER export is restored by shifting down to permissive temperature (24°C) and progressive incorporation of the two different types of cargos into the fluorescently labelled ERES can be then simultaneously captured at 3D high spatial resolution by SCLIM microscopy. By using this protocol, we have shown that newly synthesized glycosylphosphatidylinositol (GPI)-anchored proteins having a very long chain ceramide lipid moiety are clustered and sorted into specialized ERES that are distinct from those used by transmembrane secretory proteins. Furthermore, we showed that the chain length of the ceramide present in the ER membrane is critical for this sorting selectivity. Therefore, thanks to the presented method we could obtain the first direct *in vivo* evidence for lipid chain length-based protein cargo sorting into selective ERES.

## Introduction

**Fig 1:**
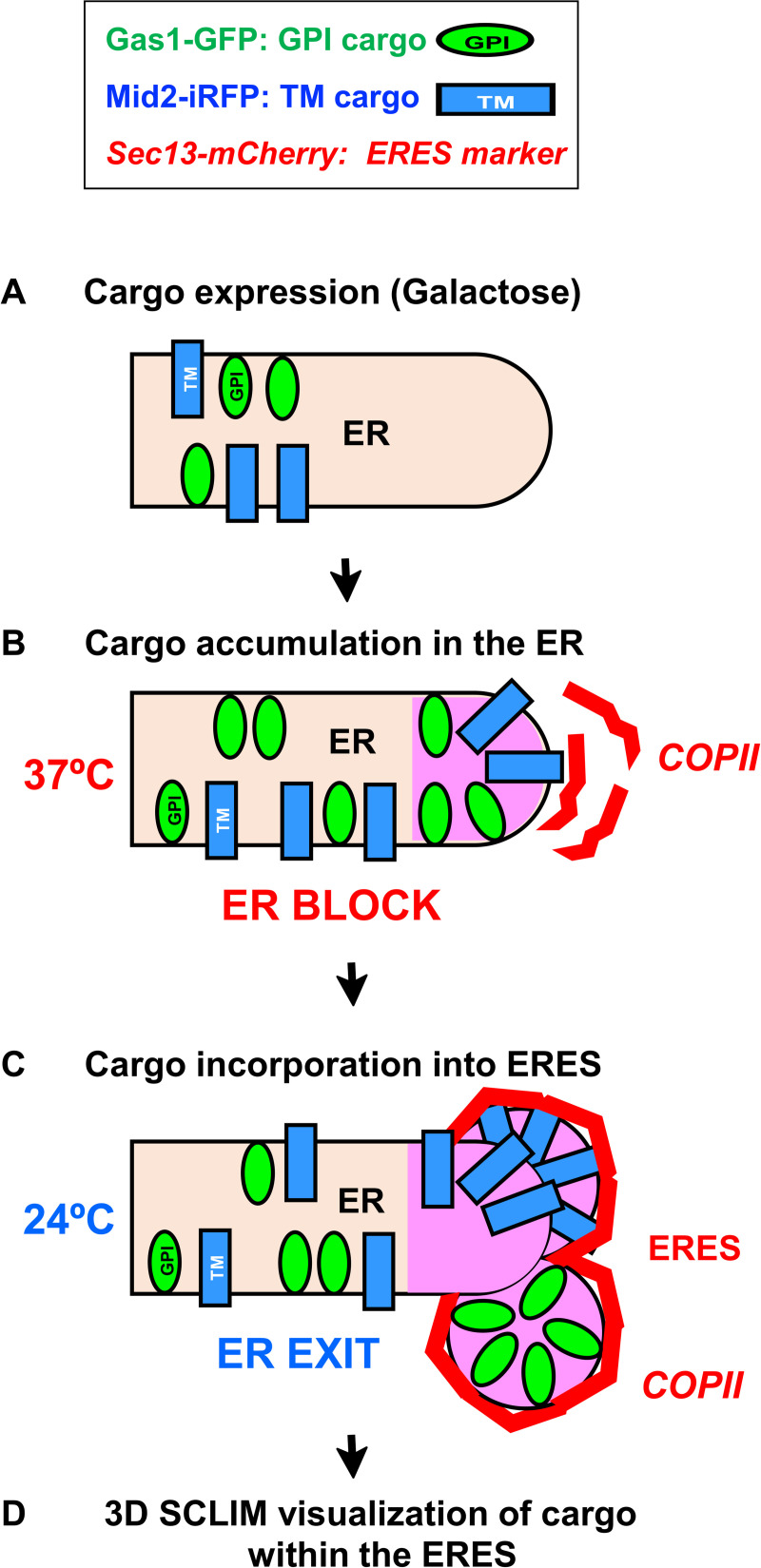
Protocol flow chart. (A) Galactose-induced co-expression of the GPI-anchored cargo Gas1-GFP (green) and the transmembrane (TM) cargo Mid2-iRFP (blue) in the *sec31-1* temperature-sensitive COPII mutant strain constitutively expressing the ERES marker Sec13-mCherry (red). (B) Accumulation of Gas1-GFP and Mid2-iRFP at the ER membrane by inhibiting COPII-coated vesicle formation at restrictive temperature (37°). (C) Restoration of ER export by shifting down to permissive temperature (24°C) and progressive incorporation of Gas1-GFP and Mid2-iRFP into the ERES. (D) Visualization of Gas1-GFP and Mid2-iRFP within the ERES by SCLIM microscopy.

Eukaryotic cells possess an elaborate endomembrane system that makes up the secretory pathway, which is responsible for the biosynthesis and delivery of one third of all proteins to their proper functional destinations and is essential for cell physiology [1]. The starting point of the secretory pathway is the endoplasmic reticulum (ER) where newly synthetized secretory proteins are incorporated into lipid vesicles that transfer them forward to the Golgi apparatus. These vesicles are generated by the sequential assembly of the cytosolic COPII coat components, which locally bends the ER membrane at specific domains called ER exit sites (ERES) [2]. In the yeast *Saccharomyces cerevisiae*, we previously reported using an *in vitro* biochemistry approach that a special class of lipid-linked cell surface proteins, glycosylphosphatidylinositol (GPI)-anchored proteins (GPI-APs), are exported from the ER to the Golgi apparatus in distinct COPII vesicles from transmembrane secretory proteins [2]. This ER sorting was addressed later using a genetic fluorescence protein tagging system imaged with conventional fluorescent microscopy [3]. Now, we have improved this protocol by combining a synchronized dual-cargo ER export system with a 3D simultaneous multi-color high-resolution live cell microscopy [4]. This improved protocol enables direct and detailed *in vivo* imaging of the simultaneous incorporation of two pairs of newly synthesized fluorescent-tagged cargos into ERES (Fig 1).

As secretory cargo proteins, we selected the GPI-AP Gas1 tagged with GFP and the transmembrane plasma membrane protein Mid2 tagged with iRFP. We expressed them under a galactose inducible promoter (GAL1) to ensure that we only observe *de novo* synthesized cargo proteins entering the ERES, which are labelled with the outer COPII coat protein Sec13, genomically tagged with mCherry. To achieve a uniform co-expression of these two GAL1-inducible cargos across the population of yeast cells, one cargo was expressed from a plasmid and the other from the genome. Gas1-GFP fusion gene was expressed under the control of the GAL1 promoter in a centromeric plasmid whereas Mid2-iRFP fusion gene under the control of the GAL1 promoter was integrated and expressed in the genome using an integrative plasmid. Furthermore, we choose the yeast genetic background W303 because has a functional GAL system (Gal+) and therefore is suitable for gene expression induction with galactose. However, live imaging of newly synthesized fluorescent-tagged proteins in the ER is still challenging because secretory proteins are continuously being synthesized and move rapidly from the ER through the secretory pathway. This implies that, at steady state, secretory cargo proteins cannot be observed at the ER because their concentration is too low for detection by fluorescence microscopy. To overcome this technical limitation, we used the thermo-sensitive COPII allele *sec31-1* as a yeast genetic tool to reversibly retain the newly synthesized fluorescent-tagged cargos in the ER in a temperature-dependent fashion [3–6]. At 37°C, the *sec31-1* mutation compromises the assembly of the COPII outer coat component Sec31 inhibiting COPII vesicle formation and ER export of cargo. Therefore, incubation of the *sec31-1* mutant strain at 37°C, leads to the retention and accumulation of galactose-induced Gas1-GFP and Mid2-iRFP at the ER, which enables their detection by fluorescence microscopy. Upon shifting down to permissive temperature (24°C), the ER export is restored and the progressive incorporation of the two different types of cargos into the ERES labelled with Sec13-mCherry can be captured and analyzed as the fluorescence signal co-localization of cargo and ERES marker by fluorescent microscopy.

To visualize the entry of newly synthesized fluorescent-tagged cargos in the ERES requires live microscopy with high spatiotemporal resolution since the ERES are very small (most of them are less than about 500 nm) and newly synthesized cargos show dynamic behavior to transiently enter into and depart from the ERES [6,7]. Indeed, although conventional epi- and confocal fluorescence microscopies can capture the movements of fluorescent-tagged cargo proteins through the secretory pathway in living cells [8], they can only achieve low spatial resolution, which is insufficient to observe cargo entry in the ERES. Super-resolution light microscopy systems like stochastic optical reconstruction microscopy (STORM), photo-activated localized microscopy (PALM), stimulated emission depletion (STED) or structured illumination microscopy (SIM), which overcome the diffraction limit of light and provide great spatial resolution, have been developed and applied to observe intracellular events. However, these systems have a low temporal resolution and therefore they are not appropriate for visualizing the dynamic behaviors of cargo in living cells. In our study we have taken advantage of a powerful microscopy technology that provides high-speed live cell imaging at high spatial resolution. This technology, termed Super-resolution confocal live imaging microscopy (SCLIM) exploits the cutting edge of the light microscopy enabling live imaging of dynamic membrane trafficking events at extremely high resolutions both in space and time [9]. It is based on the combination of a high-speed spinning-disk confocal scanner and a high-speed, high-signal-to-noise-ratio, and ultrahigh-sensitivity camera system. SCLIM is theoretically equivalent to a SIM which achieves a spatial resolution twice the diffraction limit of wide-field fluorescence microscopy (about 100 nm for GFP) [10]. In addition, because SCLIM restores the super-resolution signals by optical demodulation through the pinhole pattern of the spinning-disk confocal scanner, SCLIM enables high speed, compared to SIM, to acquire a single XY image at the maximum 30 fps, for example [10]. Thus, the SCLIM has sufficient resolution in both space and time to observe the dynamic behavior of cargo in living cells. As another technical characteristic, SCLIM is based on the spinning disk microscopy which is a multi-beam scanning method providing not only high-speed imaging but also reduced photobleaching. In addition, the image processing software Volocity can correct the photobleaching to maintain intensities during the observation time, although however, this correction is insufficient for prolonged light exposure times. To alleviate this limitation, several improvements can be implemented such as the increase of the signal of fluorescent-tagged proteins by adding more tags to the proteins or by overexpressing them.

SCLIM can also acquire simultaneous 3-color and 3D images by using an ultra-fast piezo actuator for oscillating Z-axis position of an objective lens, a magnifier lens system, 3 excitation lasers for exciting green, red, and infrared fluorescence proteins such as GFP, mCherry and iRFP, a spectroscopic unit (containing dichroic mirrors, refraction mirrors, and band-pass and long-pass filters) for separating fluorescence signals of 3 different fluorescence proteins, 3 cooled image intensifiers for amplifying the fluorescent signals, and 3 EM-CCD cameras having 512 x 512 pixels (for example, using a 100x objective lens and a 4x magnification lens, each pixel corresponds to 60 nm). The resolution of the 3D image is improved by deconvolution enhancing the super-resolution signals. Raw 3D images are deconvolved by using PSF (point-spread function) parameters of 3 fluorescence proteins optimized for the spinning-disk confocal scanner. Completely simultaneous 3D and multicolor observation is an advantage of SCLIM, and it is required for the simultaneous visualization of the incorporation of two cargos into the ERES in great detail. In a yeast cell, there are dozens of ERES, which show spherical structures and are localized on a complicated network of the ER in the 3D space inside the cell. Therefore, the SCLIM system can detect the two cargos present in the ERES by colocalization of Gas1-GFP, Mid2-iRFP and Sec13-mCherry fluorescence signals in XYZ pixels in 3D, which enables the quantification of cargo sorting into the specific ERES.

The presented assay is adaptable to a large diversity of cargos, and it also allows quantitative and real-time ER export observations. Furthermore, the combination of this assay with the power of yeast genetics to generate mutants of the ER export machinery will allow the genetic dissection of the mechanisms of ER export in an unprecedented level of detail. For instance, this assay can be used to directly address the unexplored issue of how specific cargo receptors aid to their respective cargos to enter the ERES. Finally, this method for SCLIM observation of cargo incorporation in ERES can be used in other cell systems like mammalian cells. However, temperature-sensitive mutants of the COPII coat are not available in mammalian cells. Instead, other techniques like the retention using selective hooks (RUSH) system should be used to visualize cargo present in ERES by SCLIM microscopy. This system is based on the reversible ER retention of fluorescent-tagged cargos using selective hooks that can be released from the ER upon addition of biotin [11]. Thus, synchronous release of secretory cargo from the ER could be then imaged by SCLIM.

## Materials and methods

The protocol described in this peer-reviewed article is published on protocols.io https://dx.doi.org/10.17504/protocols.io.bw82phye and is included for printing as S1 File with this article.

### Expected results

The presented protocol was used to investigate the hypothetical role of membrane lipids in protein sorting during ER export. Specifically, we addressed our model that very long acyl chain (C26) ceramide present in the ER membrane drives clustering and sorting into selective ERES of GPI-APs having C26 ceramide as lipid moiety in the yeast *Saccharomyces cerevisiae* [4]. This model proposed that both, C26 ceramides in the ER membrane and GPI-APs with C26 ceramide, would tend to coalesce into clusters or ordered ceramide-enriched domains due to the ability for self-assembly of very long chain ceramides. This C26 ceramide-dependent clustering would eventually segregate GPI-APs from transmembrane proteins and would co-sort them together with membrane ceramides into selective ERES and COPII vesicles for their subsequent co-transport to the Golgi.

We first applied the reported protocol to observe by SCLIM how the GPI-AP Gas1-GFP with C26 ceramide as lipid moiety is segregated and sorted from the transmembrane plasma membrane protein Mid2-iRFP into selective ERES upon exit from the ER. For this purpose, *sec31-1* cells expressing galactose-induced Gas1-GFP and Mid2-iRFP, and the ERES marker Sec13-mCherry were incubated at 37°C for blocking the ER export and thus accumulate newly synthesized cargos at the ER. After 30 min at 37°C the cells were immediately immobilized on a Concanavalin A-coated chamber slide and place on the microscope stage at 24°C. Upon this temperature shift down to 24°C, *sec31-1* cells are restored from the secretory block and the accumulated newly synthesized cargos start the ER export by entering the ERES. 20 min time point following the temperature shift to 24°C was selected as the optimal time to observe by SCLIM newly synthesized cargo into the ERES, since the COPII machinery has become fully functional. SCLIM imaging was performed with the acquisition speed of 4 fps for a single XY 3 color image. 31 Z slices were acquired to obtain a 3D image of 80% of a single yeast cell in 7.75 s.

**Fig 2:**
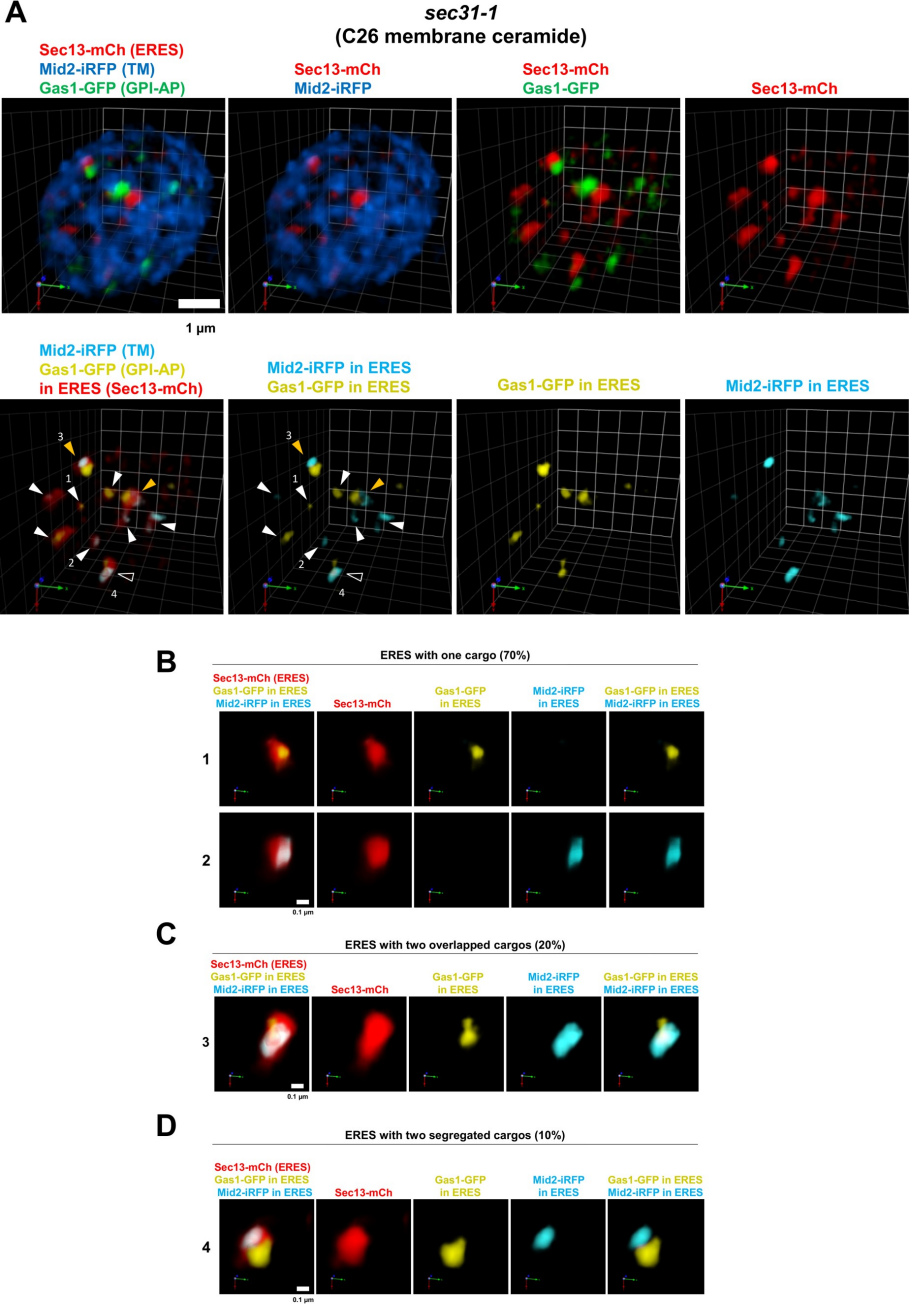
3D SCLIM visualization of the synchronized incorporation of GPI-anchored and transmembrane cargos into distinct ERES in *sec31-1* cells. *sec31-1* temperature-sensitive mutant cells co-expressing galactose-inducible secretory cargos, the GPI-AP Gas1-GFP (green) and the transmembrane (TM) protein Mid2-iRFP (blue), and the constitutive ERES marker Sec13-mCherry (red) were incubated at 37°C for 30 min and then shifted down to 24°C for releasing secretion block and imaged by SCLIM after 20 min with 4 fps as acquisition speed for a single XY 3 color image. 31 Z slices were obtained to acquire a 3D image of 80% of a single yeast cell in 7.75 s. (A) Upper panels show representative merged or individual 3D cell hemisphere images of cargos and ERES marker. Lower panels show processed images to display only the cargo (Gas1-GFP, yellow, and Mid2-iRFP, light blue) present in the ERES (red). White arrowheads: ERES with just one cargo. Open arrowheads: ERES containing two colocalizing cargos. Orange arrowheads: ERES containing two segregated cargos. Scale bar, 1 μm. Scale unit, 0.78 μm. (B, C, D) Enlarged 3D images of selected individual ERES marked in (A). (B) ERES containing only one cargo, Gas1-GFP (1) or Mid2-iRFP (2). (C) ERES containing two overlapped cargos (3). (D) ERES containing two segregated cargos in different zones (4). Scale bar, 0.1 μm.

**Fig 3:**
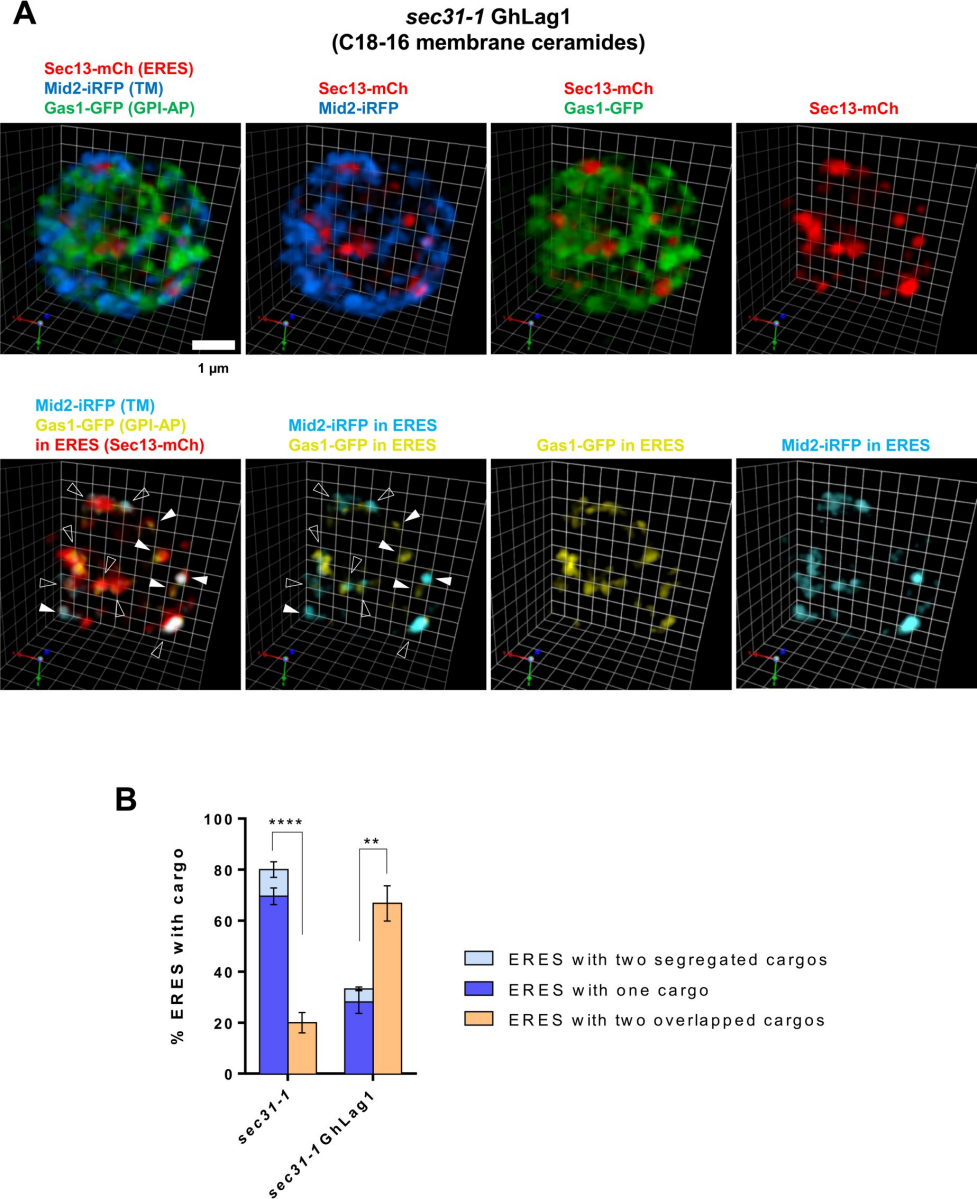
3D SCLIM visualization of the synchronized incorporation of GPI-anchored and transmembrane cargos into the same ERES in *sec31-1* GhLag1 cells. *sec31-1* GhLag1 temperature-sensitive mutant cells co-expressing galactose-inducible secretory cargos, the GPI-AP Gas1-GFP (green) and the transmembrane (TM) protein Mid2-iRFP (blue), and the constitutive ERES marker Sec13-mCherry (red) were incubated at 37°C for 30 min and then shifted down to 24°C for releasing secretion block and imaged by SCLIM after 20 min with 4 fps as acquisition speed for a single XY 3 color image. 31 Z slices were obtained to acquire a 3D image of 80% of a single yeast cell in 7.75 s. (A) Representative merged or individual 3D cell hemisphere images of cargo and ERES markers. Lower panels show processed images to display only the cargo (Gas1-GFP, yellow, and Mid2-iRFP, light blue) present in the ERES (red). White filled arrowheads: ERES containing only one cargo. Open arrowheads: ERES with overlapping cargo. Scale bar, 1 μm. Scale unit, 0.438 μm (B) Quantification of micrographs described in Figs 2A and 3A. Average percentage of ERES containing only one cargo (Gas1-GFP or Mid2-iRFP), segregated cargos, and overlapped cargos. n = 432 in 54 *sec31-1* cells in three independent experiments. n = 430 in 47 *sec31-1* GhLag1 cells in three independent experiments. Error bars = SD. Two-tailed, unpaired t test. ****P< 0.0001 (*sec31-1*) and **P = 0.004 (*sec31-1* GhLag1). Statistical significance was determined using GraphPad Prism software. For the two-tailed Student’s t-test, differences among groups were considered significant for P < 0.05 (*).

3D observation by SCLIM after 20 min at 24°C showed that most of Gas1-GFP and Mid2-iRFP still accumulate at the ER since they display the typical ER-like pattern. Nevertheless, Gas1-GFP and Mid2-iRFP were differently localized in the ER. While Mid2-iRFP was distributed throughout the ER membrane, Gas1-GFP was concentrated in clusters at discrete regions of the ER membrane (Fig 2A). Interestingly, Gas1-GFP clusters were often found adjacent ERES labelled with Sec13-mCherry (< 100nm). Next, we addressed whether ER accumulated Gas1-GFP and Mid2-iRFP are sorted into different ERES. We used SCLIM microscopy for 3D and triple co-localization of Gas1-GFP, Mid2-iRFP and Sec13-mCherry (Fig 2 and S1 Movie). This 3D co-localization analysis allowed us to quantify the proportion of ERES in which only one type of cargo or both are present (Fig 3B). For the statistical analysis, we count 432 ERES in 54 cells in three independent experiments. We found that most of the ERES (70%) exclusively contained only one type of cargo. Fig 2B shows representative examples of ERES with only Gas1-GFP (1) or only Mid2-iRFP (2). In contrast, only about 20% of ERES contained both cargos overlapping in the same area. Fig 2C shows a representative example of ERES with overlapping Gas1-GFP and Mid2-GFP (3). Interestingly, some ERES (10%) were found containing both cargos, but they segregated in clearly distinct zones. Fig 2D shows a typical example of cargos segregated in distinct areas of the same ERES [4]. Therefore, these results indicate that Gas1-GFP is sorted into different ERES than Mid2-iRFP upon ER exit.

Next, we applied this assay to test our previous hypothesis that very long acyl chain (C26) ceramide present in the ER membrane drives the specific clustering and sorting of GPI-APs into selective ERES. For this purpose, we took advantage of the genetically modified yeast strain GhLag1, which synthesizes shorter membrane ceramides (C18-C16) than the wild-type strain (C26), but still expresses Gas1-GFP having C26 ceramide, the same very long chain GPI-lipid as in wild type [4]. Therefore, to specifically address the hypothetical role of the acyl chain length of membrane ceramides in ER clustering and sorting, we conducted 3D SCLIM visualization of Gas1-GFP and Mid2-iRFP in GhLag1 strain bearing the *sec31-1* thermo-sensitive mutant allele (Fig 3A and S2 Movie). *sec31-1* GhLag1 cells expressing Gas1-GFP, Mid2-iRFP and Sec13-mCherry were incubated at 37°C for 30 min, shifted down to 24°C for recovery of ER export and imaged by SCLIM after 20 min. We observed that most of newly synthesized Gas1-GFP was unclustered and distributed throughout the ER membrane. Next, we performed the 3D and triple co-localization analysis to quantify the percentage of ERES in which only one type of cargo or both are included (Fig 3). For the statistical analysis, we count 430 ERES in 47 *sec31-1* GhLag1 cells in three independent experiments. As shown in Fig 3B, compared with the *sec31-1* strain, the *sec31-1* GhLag1 strain exhibited a larger percentage of ERES (67%) with both cargos overlapping within them. This data shows that in *sec31-1* GhLag1 cells having shorter C18-C16 membrane ceramides, Gas1-GFP is rerouted and enter the same ERES as transmembrane cargo. Therefore, decreasing the acyl chain length of membrane ceramide from C26 to C18-C16 disrupts Gas1-GFP sorting into selective ERES. By taking advantage of the presented protocol, we demonstrate that ceramide acyl chain length in the ER membrane is an essential determinant for GPI-AP clustering and sorting from the ER in yeast.

## Supporting information

S1 File Step-by-step protocol, also available on protocols.io. (PDF)

S1 Movie Multi-angle 3D reconstructed movie representing sorting of Gas1-GFP (green) and Mid2-iRFP (blue) into different ERES (red) in *sec31-1* cells. (MP4)

S2 Movie Multi-angle 3D reconstructed movie representing incorporation of Gas1-GFP (green) and Mid2-iRFP (blue) into the same ERES (red) in *sec31-1* Ghlag1 cells. (MP4)
