# Supplementary material for: Assay for dual cargo sorting into endoplasmic reticulum exit sites imaged by 3D Super-resolution Confocal Live Imaging Microscopy (SCLIM)
Source: PLoS One. 2021 Oct 1;16(10):e0258111. doi: 10.1371/journal.pone.0258111 (PMC8486111; doi:10.1371/journal.pone.0258111)
Supplement: S1 File — (PDF) [file pone.0258111.s001.pdf]

Assay for dual cargo sorting into endoplasmic reticulum exit sites imaged by Super-resolution Confocal Live Imaging Microscopy (SCLIM)

Sofia Rodriguez-Gallardo †[1], Kazuo Kurokawa †*[2], Susana Sabido-Bozo[1], Alejandro Cortes-Gomez[1], Ana Maria Perez-Linero[1], Auxiliadora Aguilera-Romero[1], Sergio Lopez[1], Miho Waga[2], Akihiko Nakano[2], Manuel Muñiz[1]

[1]Department of Cell Biology, University of Seville and Instituto de Biomedicina de Sevilla (IBiS), Hospital Universitario Virgen del Rocío/CSIC/Universidad de Sevilla, 41012, Seville, Spain.;

[2]Live Cell Super-Resolution Imaging Research Team, RIKEN Center for Advanced Photonics, Saitama, Japan

1 Works for me

Manuel Muniz

ABSTRACT

Understanding how in eukaryotic cells thousands of proteins are sorted from each other through the secretory pathway and delivered to their correct destinations is a central issue of cell biology. We have further investigated in yeast how two distinct types of cargo proteins are sorted into different endoplasmic reticulum (ER) exit sites (ERES) for their differential ER export to the Golgi apparatus. We used an optimized protocol that combines a live cell dual-cargo ER export system with a 3D simultaneous multi-color high-resolution live cell microscopy called Super-resolution Confocal Live Imaging Microscopy (SCLIM). Here, we describe this protocol, which is based on the reversible ER retention of two *de novo* co-expressed cargos by blocking COPII function upon incubation of the thermo-sensitive COPII allele *sec31-1* at restrictive temperature (37ºC). ER export is restored by shifting down to permissive temperature (24ºC) and progressive incorporation of the two different types of cargos into the fluorescently labeled ERES can be then simultaneously captured at 3D high spatial resolution by SCLIM microscopy. By using this protocol, we have shown that newly synthesized glycosylphosphatidylinositol (GPI)-anchored proteins having a very long chain ceramide lipid moiety are clustered and sorted into specialized ERES that are distinct from those used by transmembrane secretory proteins. Furthermore, we showed that the chain length of the ceramide is critical for this sorting selectivity. Therefore, thanks to the presented method we could obtain the first direct in vivo evidence for lipid chain length-based protein cargo sorting into selective ERES.

PROTOCOL INFO

Sofia Rodriguez-Gallardo †, Kazuo Kurokawa †*, Susana Sabido-Bozo, Alejandro Cortes-Gomez, Ana Maria Perez-Linero, Auxiliadora Aguilera-Romero, Sergio Lopez, Miho Waga, Akihiko Nakano, Manuel Muñiz . Assay for dual cargo sorting into endoplasmic reticulum exit sites imaged by Super-resolution Confocal Live Imaging Microscopy (SCLIM). **protocols.io**
https://protocols.io/view/assay-for-dual-cargo-sorting-into-endoplasmic-reti-bw82phye

KEYWORDS

Endoplasmic reticulum exit sites, COPII coat, GPI-anchored protein cargo, transmembrane secretory cargo, Super-resolution live cell imaging, Simultaneous 3-color visualization

CREATED

Aug 09, 2021

LAST MODIFIED

Sep 16, 2021

PROTOCOL INTEGER ID

52218

Materials

1. Budding yeast cells (W303) with the *sec31-1* thermosensitive mutant allele expressing two galactose-inducible cargos and the constitutive COPII protein as ERES marker tagged with different fluorescent proteins. This yeast strain can be obtained upon request from our laboratory.
2. Toothpicks sterilized before use (any brand).
3. Yeast extract (Pronadisa catalog number: 1702.00).
4. Peptone (Panreac catalog number: A2210,0500)
4. Peptone (Panreac catalog number: A2210,0500).).
6. Raffinose (Panreac catalog number: A6882,0100).
7. Galactose (Panreac catalog number: A1131,1000).
6. Yeast nitrogen base without amino acids (Difco Laboratories, catalog number: 291940).
7. Amino acids and bases (Uracil SIGMA catalog number: U0750, L-Leucine SIGMA catalog number: L8000, L-Tryptophan SIGMA catalog number: T0254, Adenine SIGMA catalog number: A8626, L-Histidine SIGMA catalog number: H8000, L(+)-Lysine SIGMA catalog number: L5501, L-Methionine SIGMA catalog number: M9625)
8. Agar (Oxoid catalog number: LP0011)..
9. Sterile 5 ml tubes (any brand).
10. 1.5 ml microcentrifuge tubes (any brand).
11. Pipette tips, sterile (Sorenson, 1-200 µl Graduated tip, catalog number: 27730).
12. 8 well chamber slide (Ibidi, catalog number: 80821).
13. Concanavalin A (Sigma-Aldrich, catalog number: C2010).

Recipes

1. SD agar plates: synthetic minimal medium containing 0.67% yeast nitrogen base, 0.5% ammonium sulfate and with 2% bacteriological agar, supplemented with the appropriate amino acids and bases as nutritional requirements and containing 2% glucose.
2. YPD medium: 1% yeast extract, 2% peptone, supplemented with 0.2% adenine and uracil, and containing 2% glucose.
3. YPR medium: YPD medium: 1% yeast extract, 2% peptone, supplemented with 0.2% adenine and uracil, and containing 2% raffinose.
4. YPG medium: 1% yeast extract, 2% peptone, supplemented with 0.2% adenine and uracil, and containing 2% galactose.
5. SG medium: synthetic minimal medium containing 0.67% yeast nitrogen base, 0.5% ammonium sulfate, supplemented with the appropriate amino acids and bases as nutritional requirements and containing 2% galactose.
6. Concanavalin A fresh working solution (0.2 mg/ml). Dissolve Concanavalin A in distilled water immediately before use.

Equipment

1. Incubator shaker (Eppendorf, model M1299-0092).
2. Micropipettes (Gilson, Pipetman, models: P20, P200, P1000).
3. Spectrophotometer (any brand with 595 nm wavelength)
2. Thermoshaker (Thermo Fisher, model 13687711).
4. SCLIM is equipped with:
4.1. Inverted fluorescence microscope (Olympus, model: IX-73)
4.2. Objective lens (Olympus, UPlanSApo 100x NA 1.4 oil objective lens).
4.3. Spinning-disk confocal scanner (Yokogawa Electric, CSU10 custom made model). Other type of CSU can be used (e.g., CSU10, CSUX1, CSUW1). Confocal scanner is equipped with custom made dichroic mirror having high spectroscopic properties for 3-color observation (Kurokawa et al., 2013).
4.4. Excitation lasers: 3 solid-state lasers with emission at 473 nm (Cobolt, CW 473nm, DPSS, 50 mW), 561 nm (Cobolt, CW 561 nm, DPSS, 50 mW), and 671 nm (CrystaLaser, CW 671 nm, DPSS, 100 mW). These 3 lasers can excite GFP, mRFP or mCherry, and iRFP simultaneously. Irradiation of the sample by these 3 lasers is controlled by 3 independent electromagnetic shutters (Sigma Koki). These shutters are operated by the demand of the custom made software. Irradiation powers of 3 lasers can be adjusted with variable ND filters.
4.5. Custom-made spectroscopic unit, image intensifiers (Hamamatsu Photonics) equipped with a custom-made cooling system, magnification lens system for giving 266.7× final magnification.
4.6. EM-CCD cameras (Hamamatsu Photonics, model, C9100-13).
4.7. Piezo actuator (Yokogawa Electric, custom made model).
4.8. Thermo-control stage (Tokai Hit)
4.9. Image acquisition is executed by custom-made software (Yokogawa Electric).
4.10. Image processing by deconvolution with Volocity software (Perkin Elmer).

Software

1. Volocity software (PerkinElmer).
2. GraphPad Prism.

Yeast growth and culture

1 Pick up *sec31-1* temperature-sensitive mutant yeast cells expressing two galactose-inducible secretory cargos (i.e. the GPI-AP cargo Gas1-GFP and the transmembrane plasma membrane protein Mid2-iRFP) and the constitutive COPII outer coat protein Sec13-mCherry as ERES marker from a frozen stock using a sterile toothpick, streak yeast cells on a SD agar plate with appropriate amino acid supplements and incubate them at 24 °C for 2-3 days.

2 Inoculate yeast cells into 3 ml YPD medium in a 5 ml sterile tube and grow them for 6-8 h to 0.5 x 10^7 cells/ml at 24°C with shaking at 500 rpm.

3 Pellet yeast cells again by centrifugation at 3000 x g for 5 min, discard the supernatant, resuspend in 3 ml of YPR and grow them to 0.4 x 10^7 cells/ml overnight at 24°C with shaking at 500 rpm.

4 The next day, pellet yeast cells by centrifugation at 3000 x g for 5 min, discard the supernatant and resuspend in 4 ml of YPG medium.

5 Pellet yeast cells again by centrifugation at 3000 x g for 5 min, discard the supernatant, resuspend in 3 ml of YPG and incubate at 24 °C for 1 h to induce galactose-regulated expression.

6 Pellet yeast cells by centrifugation at 3000 x g for 5 min, discard the supernatant, resuspend in 1 ml of SG medium and transfer to a 1.5 ml tube.

7 Pellet yeast cells again by centrifugation at 3000 x g for 5 min, resuspend in 1ml of SG medium and incubate at 37 °C for 30 min in a thermoshaker to accumulate galactose-induced protein cargos in the ER by inhibiting COPII-coated vesicle formation.

Yeast sample preparation

8 During the 30 min incubation at 37°C in SG (step 7), prepare a fresh Concanavalin A-coated well chamber slide to immobilize the yeast cells for microscopic observation.

8.1 Add 25 µl of 0.2 mg/ml Concanavalin A to the wells of the chamber slide for about 2 min.

8.2 Aspirate the excess Concanavalin A solution.

8.3 Let the Concanavalin A dry at room temperature for about 10 min.

9 After incubation for 30 min at 37°C in SG (step 7), apply about 100 µl of yeast liquid culture into a well of the Concanavalin A-coated chamber slide at room temperature (this is the start of the temperature shift to 24ºC).

10 Wait for 2 min for immobilization of yeast cells on the Concanavalin A-coated chamber slide.

11 Place the Concanavalin A-coated chamber slide with immobilized yeast cells on the microscope stage, which has been already pre-warmed at 24°C (permissive temperature) by an automatic thermo-control system. Yeast cells are now ready for live imaging by 3D SCLIM (step13). We have selected 20 min time point following the temperature shift to 24ºC (step 9) as the optimal time to observe by 3D SCLIM newly synthesized cargo into the ERES, as the COPII machinery has become fully functional.

Live cell imaging by 3D Super-resolution Confocal Live Imaging Microscopy (SCLIM)

12 Set up of SCLIM before observation.

Due to several factors, such as chromatic aberrations of lenses in the whole microscopic and spectroscopic system, the 3 different fluorescence signals coming from the same position (XYZ) may be projected to different positions (XYZ) of 3 cameras. To conduct simultaneous 3-color 3D observation, it is necessary first to check and correct the XYZ in 3 alignments of these fluorescence signals by aligning the light spots from each of the 3 cameras.

12.1 Stop the rotation of the spinning-disk of the confocal scanner.

12.2 Apply a weak light from a halogen lamp light source to objective lens.

12.3 Obtain the halogen light images passed through the pinhole with 3 cameras.
Note: images with practically the same intensities in 3 cameras can be acquired since the gain of amplification in 3 Image intensifiers are controlled independently.

12.4 Adjust the angles of dichroic millers and reflection millers of the custom-made spectroscopic unit to correct the XY positions of the images of the 3 cameras.

13 Image acquisition

Fluorescent proteins have a wide range of emission wavelengths. Thus, for example, when GFP is expressed at a very high level, the GFP fluorescence signal can be detected even by a camera with a longer-wavelength emission filer. Such leakage of fluorescence signals is called bleed-through or crosstalk. It is critical to avoid such bleed-through signals as much as possible. The powers of 3 excitation lasers and the amplification gains of 3 image intensifiers should be independently controlled to minimize bleed-through.

13.1 Observe live yeast cells expressing cargos (Galactose-inducible Gas1-GFP and Mid2-iRFP) and ERES marker (Sec13-mCherry) by SCLIM. In these cells, we can observe that a small portion of both cargos enters into different ERES.

13.2 2D confocal fluorescence data are separated in the custom-made spectroscopic unit. GFP, mCherry, and iRFP fluorescence images finally pass through the spectral windows defined by 2 band pass filters (GFP; 490-545 nm, and mCherry; 580-660 nm) and 1 long pass filter (iRFP; 680 nm-) and are simultaneously collected by synchronized 3 EMCCD cameras.

13.3 Collect Z slice of 3-channel images. An appropriate range of Z-axis (e.g., 3 µm cover about 80% of yeast cell volume) is scanned into optical slices 100 nm apart by the piezo actuator equipped to the objective lens (ie. 31 Z stacks per 3 µm deep cell, each Z stack has a thickness of 100 nm). Each XY 3 color image is captured in 0.25 s (4 fps as acquisition speed).

13.4 Raw data sets (Z slice 3-channel images) are once stored in the TIFF format in the hard-disk memory of the image acquisition computer.

3D image reconstruction and deconvolution using Volocity software

14 Transfer raw data sets to the computer installed with Volocity software.

15 Reconstruct 3D images from raw data sets using Volocity software.

15.1 Select the option "Create new" from Actions menu in Volocity to make the Image sequence.

15.2 Raw data sets in 3 channels are read into Image sequence and the 3D images in 3 channels are reconstructed.

15.3 Select the option "Remove Noise" from the Tools menu and remove noise from all 3D images using a fine filter.

15.4 Select the option "Change Colors" from the Tools menu and change thecolors of 3 channels (e.g., GFP as green, mCherry as red, and iRFP as blue).

15.5 Select the option “Correct Photobleaching” from the Tools menu and correct for photobleaching of 3D images to maintain intensities that are reduced by photobleaching during the observation time.

15.6 Select the option "Properties" from the Edit menu and change information of XYZ pixel sizes in 3D images. Pixels of (X) and (Y) are 0.06 µm and pixel of (Z) is 0.1 µm when we use 100x objective lens and 4x amplification lens and scan Z-axis with optical slices 100 nm apart.

Analysis and presentation of 3D images

16 Select manually regions of interest in deconvolved 3D images. Present the images as XYZ mode and outline the region of interest using the selection tool. Select the option "Crop to Selection" from the Actions menu to create a new deconvolved 3D image. Present 3D images shown in 3D opacity mode at any desired angles.

17 Perform measurement of "ERES including cargo" by colocalization analysis with automatic thresholding of Volocity software. Care should be taken when applying automatic thresholding values for image analysis. Ensure that thresholds are maintained at equal values through whole sets of data.

18 Statistical analysis

Calculate the average percentage of ERES containing only one cargo (Gas1-GFP or Mid2-iRFP), segregated cargos, and overlapped cargos. Count more than 400 ERES in about 50 *sec31-1* cells in three independent experiments. Determine the statistical significance by using GraphPad Prism software. For the two-tailed student's t-test, consider $P < 0.05$ (*) as significant differences among groups.
